# Targeting glucocorticoid receptor signaling pathway for treatment of stress-related brain disorders

**DOI:** 10.1007/s43440-024-00654-w

**Published:** 2024-10-03

**Authors:** Tansu Göver, Michal Slezak

**Affiliations:** 1 Lukasiewicz Research Network - PORT Polish Center for Technology Development, ul. Stabłowicka 147, 54-066 Wroclaw, Poland; 2 Department of Biophysics and Neuroscience, Wroclaw Medical University, ul. Chałubińskiego 3A, 50-368 Wroclaw, Poland

**Keywords:** HPA axis, Glucocorticoid receptor, Stress, Major depression, Depressive-like behavior, Anxiety, FKBP51, PROTAC

## Abstract

The hypothalamic–pituitary–adrenal (HPA) axis plays a central role in governing stress-related disorders such as major depressive disorder (MDD), anxiety, and post-traumatic stress disorder. Chronic stress or early life trauma, known risk factors of disease, alter HPA axis activity and pattern of glucocorticoid (GC) secretion. These changes have consequences for physiological processes controlled by glucocorticoid receptor (GR) signaling, such as immune response and metabolism. In the brain, the aberrant GR signaling translates to altered behavior, making the GR pathway a viable target for therapies of stress-related disorders. One of the crucial elements of the pathway is FKBP5, a regulator of GR sensitivity and feedback control within the HPA axis, in which genetic variants were shown to moderate the risk of developing psychiatric conditions. The difficulty in targeting the GR-FKBP5 pathway stems from tailoring the intervention to specific brain regions and cell types, in the context of personalized genetic variations in GR and GR-associated genes, like *FKBP*5. The development of selective inhibitors, antagonists, and approaches based on targeted protein degradation offer insights into mechanistic aspects of disease and pave the way for improved therapy. These strategies can be employed either independently or in conjunction with conventional medications. Concomitant advancements in personalized drug screening (e.g. in vitro models exploiting induced pluripotent stem cells, iPSCs) bring the potential for optimization of therapy aiming to rescue central deficits originating from the HPA imbalance. In this mini-review, we discuss potential therapeutic strategies targeting GR signaling in stress-related disorders, with a focus on personalized approaches and advancements in drug development.

## Introduction

Stress constitutes a complex psychological and physiological reaction to challenging or threatening stimuli, disrupting an individual's homeostasis [1]. This multifaceted response is orchestrated by the activation of the hypothalamic–pituitary–adrenal (HPA) axis, culminating in excessive secretion of GCs to mobilize energy resources, regulate immune response and cognitive functions, believed to act to re-establish homeostasis and promote stress memory [2, 3]. Stress-induced increase in plasma GCs activates the GR and results in tissue- and cell-type specific transcriptional effects. The last decades revealed multiple mechanisms regulating GR sensitivity, including genetic and environmental factors affecting GR expression, dysregulation of key components of the GR signaling pathway, or changes in the local metabolism of GCs. Genetic polymorphisms in the *NR3C1* gene, encoding GR, constitute a primary regulatory mechanism of GR function and expression [4]. The effects of environmental factors depend on the individuals’ history and genetic predisposition. Chronic stress, a major risk factor for many psychiatric disorders, leads to prolonged exposure to high levels of GCs, which, through homeostatic regulation, results in decreased expression of GR and reduced feedback inhibition of the HPA axis [5]. Likewise, certain pharmacological compounds, such as synthetic GCs, antipsychotics, or chemotherapeutic agents, can downregulate GR expression or sensitivity, by interfering with signaling pathways [6–8]. The HPA axis dysfunction is a shared feature of psychiatric disorders, including anxiety disorders, PTSD, or MDD, where glucocorticoid resistance is one the most frequent physiological symptoms [9, 10]. Since GR signaling is tightly regulated at the level of individual organs in a cell-specific fashion [11], targeted efforts are being sought to counteract the impaired responsiveness of the GR pathway for restoring tissue homeostasis. The task is particularly challenging in the context of brain circuits governing behavior, due to ubiquitous expression of the GR across multiple brain regions and cell types. In this mini-review, we discuss recent approaches targeting the GR signaling pathway, with an emphasis of exploiting these developments for neural cells as a strategy for alleviating symptoms of mood disorders.

## GR–FKBP5 pathway and mood disorders

**Fig. 1:**
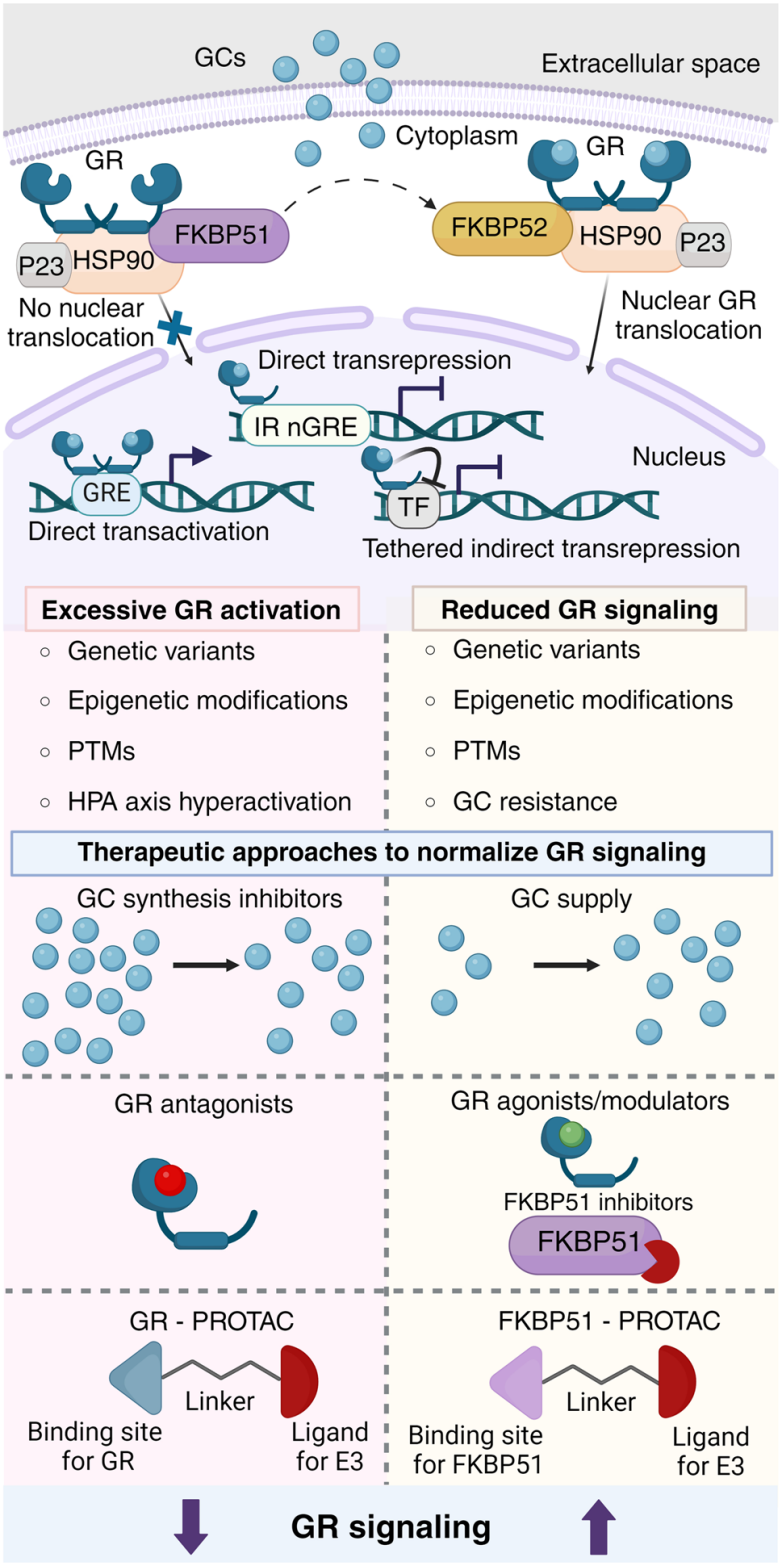
Strategies for rescuing impaired GR signaling. In physiological conditions, chaperone proteins form a complex that maintains the GR in a high-affinity state in the absence of ligands. Upon GC binding, FKBP51 is replaced by FKBP52, facilitating the translocation of the receptor to the nucleus where GR regulates gene expression by binding to (+)GREs, IR nGREs, or through transrepression mechanisms. Genetic or environmental risk factors may affect GR-dependent gene expression dynamics. Therapeutic approaches to mitigate excessive GR activation (left) include the use of GC synthesis inhibitors, GR antagonists or PROTACs designed to degrade GR. Strategies for decreased GR activation (right) include GC replacement therapy, the use of agents that enhance GR function, like agonists and modulators, or indirectly, through functional inhibition of FKBP51. *GC* glucocorticoid, *GR* glucocorticoid receptor, *FKBP51* FK506-binding protein 51, *FKBP52* FK506-binding protein 52, *HSP90* heat shock protein 90, *P23* co-chaperone protein p23, *(+)GRE* positive glucocorticoid response elements, *IR nGRE* inverted repeats negative glucocorticoid response elements, *HPA* hypothalamic–pituitary–adrenal, *PTM* post-translational modifications, *PROTAC* proteolysis targeting chimera, *TF* transcription factor

Physiologically, GCs are secreted from adrenals under tight control of the circadian system, reaching peak concentration in the plasma before the onset of the active phase [12], fine-tuned by complex ultradian fluctuations in GC dynamics [13]. GCs act through the mineralocorticoid receptor (MR) and GR, both being members of the nuclear receptor superfamily, with the nuanced interplay between the two shaping physiological processes and stress-related pathologies [14]. MR binds GCs with much higher affinity leading to permanent receptor occupation, therefore GR is believed to be activated by elevated GCs, either during circadian peak or upon stress [15]. In a ligand-free state, GR resides in the cytoplasm in a protein complex, with FKBP51, HSP90, and p23 being essential to maintain the GR complex in its high-affinity form [16–18]. When GCs bind to GR, FKBP51 is replaced with FKBP52 enabling the receptor translocation from the cytoplasm to the nucleus, required for its transcriptional activity [19]. GR can activate or repress target gene transcription by binding to positive GC response elements ((+)GREs, [20]), inverted repeats negative GC response elements (IR nGREs, [21]) or through tethered indirect transrepression [22] (Fig. 1). Together with modulatory effect on other transcription factors [23], GR activation exerts control over a transcriptional program regulating energy metabolism, immune response and other homeostatic processes [24, 25].

The co-chaperone protein FKBP51 (FK506-binding protein 51, encoded by the *FKBP5* gene) is a crucial modulator of GR transcriptional activity by diminishing ligand binding and delaying GR nuclear translocation [26–28]. FKBP51 itself is a direct transcriptional target of GR and this activation forms a basis for an ultrafast feedback mechanism, essential for preventing overstimulation of GR signaling and ensuring a balanced stress response [29]. FKBP51 is a pivotal regulator of the HPA axis, modulating GR sensitivity and thereby maintaining cortisol homeostasis, which is crucial in the pathophysiology of stress-related disorders [30]. Genetic polymorphisms in *FKBP5* disrupt the negative feedback mechanism of the HPA axis, resulting in prolonged cortisol exposure and increased susceptibility to psychiatric disorders such as PTSD, MDD, and anxiety disorders [31].

Dysfunctions in HPA axis activity are commonly observed in patients with mental disorders and a close association between the regulation of stress response and the onset or worsening of depressive symptoms is well documented [32–34]. In line with these observations, the GR-dependent gene network was shown to shape individuals’ susceptibility to disease and predict the outcome of antidepressant therapy [35, 36]. Likewise, polymorphisms in *FKBP5* were shown to modulate individuals’ stress response and predisposition to developing psychiatric symptoms [37–39] and epigenetic modifications of *FKBP5* were described as a signature of an early life trauma [40], with the effect on the adult stress response [41–43]. Since these phenotypes are consistent between mice to humans, therapeutic approaches aiming to normalize the stress response and rescue discrete molecular and behavioral symptoms by acting on the GR pathway are discussed below in the context of translational research [44].

The relationship between GR levels and psychiatric symptoms is complex and context-dependent [45, 46]. Higher GR levels may increase sensitivity to GCs, potentially leading to enhanced stress responses and a predisposition to depressive symptoms [47]. Elevated GC levels have been linked to changes in the brain structure and function, including alterations in neurotransmitter systems implicated in mood regulation [48]. Individuals with Cushing’s syndrome, characterized by hypercortisolism, frequently experience depression and anxiety [49]. In rodent models, chronic exposure to corticosterone (CORT) induces depressive-like molecular and behavioral phenotypes, analogous to those observed for example in MDD [50–52]. MDD is characterized by the presence of psychological (persistent low mood, anhedonia, loss of interest in daily activities), as well as physiological symptoms, the latter encompassing multiple processes regulated by GCs, e.g. metabolic control (symptom: quick weight loss or gain) or daily rhythms (symptom: insomnia or hypersomnia). These symptoms may relate to simultaneous elevated expression of GR and lowered of FKBP5, which can be monitored as a biomarker in the blood of patients with MDD [47]. Chronic or early life stress, both considered major risk factors for MDD [53], are known to permanently affect the HPA axis functioning and sustainably elevate plasma GCs. Therefore, therapeutic strategies aiming to reduce the excessive GR signaling are assumed beneficial through limiting negative consequences of GR hyperactivation.

Conversely, decreased GR levels reduce the sensitivity to GCs. Glucocorticoid resistance may originate from mutations or genetic variants in crucial genes of GR pathway as a primary cause, or result from excessive GC signaling leading to homeostatic downregulation of GR [54]. In the HPA axis, GC resistance results in limited feedback inhibition and HPA overactivation [55]. In turn, in target tissues, such as the central nervous system (CNS), the GC resistance leads to decreased homeostatic control of physiological processes, with potential consequences for brain circuits controlling behavior [32]. The reduction of GR expression was consistently reported in the brains of patients with MDD or suicide cases [56–58], as well as in the animal models of chronic stress and early life trauma [59, 60]. Here, therapeutic strategies aim to enhance GR signaling to restore physiological responsiveness of the GR. The support for this concept was obtained with antidepressants. Clomipramine, a tricyclic antidepressant, was shown to inhibit FKBP51 SUMOylation, previously shown as enhanced under conditions of cellular stress [61]. The inhibition resulted in lowering FKBP51 affinity to GR, which facilitated the recruitment of FKBP52, eventually improving the biological function of the GR [62]. In another study, chronic corticosterone (CORT) treatment in mice led to a decrease in hippocampal GR protein levels. Remarkably, a single dose of ketamine, an NMDA receptor antagonist with demonstrated rapid and sustained antidepressant responses, but not fluoxetine, a selective serotonin reuptake inhibitor (SSRI), reversed this phenotype, along with rescuing dendritic plasticity [63]. These findings suggest that the GR-FKBP51 complex may play a crucial role in antidepressants action, encouraging further studies of direct manipulation of the pathway.

Overall, the optimal GR levels likely involve a delicate balance, where adequate receptor levels and proper functioning are necessary to maintain physiological homeostasis and resilience to stress. However, deviations from this balance, whether towards higher or lower GR levels, can contribute to the development or exacerbation of depressive symptoms [34, 64]. Therapeutic strategies are therefore being explored aiming to normalize GR signaling and stress response.

## Strategies for attenuating excessive GR activation

### GC synthesis inhibitors

GC synthesis inhibitors, such as metyrapone or ketoconazole, block key enzymes involved in the biosynthesis of GCs (e.g. metyrapone effectively inhibits the 11-beta-hydroxylase, which catalyzes the final step in cortisol synthesis) [65, 66]. These compounds are used therapeutically to manage conditions characterized by hypercortisolism, e.g. Cushing's syndrome, and are being explored as potential antidepressants due to their demonstrated ability to modulate the stress response system [32, 34, 67]. For example, the addition of metyrapone hastened the onset of antidepressant effects in MDD patients [68], while high-dose ketoconazole reduced anxious behaviors and lowered levels of CORT in a rat model of chronic post-traumatic anxiety [69]. However, long-term use of these drugs may result in adrenal insufficiency, electrolyte imbalances, disturbances in glucose metabolism, compensatory hyperactivation of HPA and adrenals hypertrophy [65]. It is also worth noting that individual responses to these medications can vary and further research is needed to fully elucidate their efficacy and safety profiles in the management of mood disorders.

### MR antagonists

Commonly used MR antagonists, such as spironolactone and eplerenone, are primarily used to treat conditions such as hypertension and heart failure. The mechanism includes a competitive block of aldosterone binding to MR, thus hindering the reabsorption of sodium and chloride ions [70]. Spironolactone was shown to prevent the induction of depressive-like behavior in a mouse model of chronic CORT administration [71]. The mechanism of this action is unclear and may relate to GR-MR interactions or the impact of MR on modulating the HPA axis [72]. Recently, non-steroidal MR antagonists, like finerenone, have been developed to provide high selectivity for MR while minimizing sex steroid-related side effects and demonstrating significant cardiorenal benefits [73]. While no reports exist on the impact of those compounds on depressive-like phenotypes, their high selectivity is a promising feature for resolving the contribution of MR to discrete symptoms of the psychiatric spectrum [14].

### GR antagonists

There are presently no medications that directly target the GR for psychiatric purposes [74]. Mifepristone (also known as RU-486), a progesterone receptor antagonist displaying also high affinity to GR, was shown to reduce depressive symptoms in patients with psychotic depression and bipolar disorder [75, 76], however serious side effects associated with long-term treatment are discouraging for therapeutic use in psychiatry [65]. Preclinical research suggests that recently developed non-steroidal GR antagonists, such as CORT125134, which accomplished phase III clinical trial for Cushing’s syndrome, hold therapeutic potential for the treatment of diseases associated with GR overactivation [77]. Of note, the effects of GR antagonists might modulate alternative signaling pathways or gene expression profiles to and should be carefully examined over time for meaningful interpretation [78].

### GR-PROTAC

Traditional GR antagonists can be ineffective due to genetic variants of the receptor or alterations in the signaling pathway, which can also impact other steroid hormone receptors, leading to undesirable side effects [79, 80]. Alternative mode for functional elimination of the receptor is targeted protein degradation (TPD) [81], encompassing strategies such as Proteolysis Targeting Chimera (PROTAC) [82], Lysosome-Targeting Chimera (LYTAC) [83], antibody-based PROTACs [84], Trim-Away [85], and specific and non-genetic inhibitors of apoptosis protein-dependent protein erasers (SNIPERs) [86–88]. The PROTAC technology is based on the use of a bifunctional molecule, where one part selectively recognizes a target protein, while the second domain recruits an E3 ubiquitin ligase, leading to target ubiquitination and subsequent degradation by the proteasome [89]. This technology offers advantages over traditional inhibition methods by providing a more complete and durable suppression of target proteins. PROTACs have shown promise in various therapeutic areas, including oncology [90, 91] and neurodegenerative diseases [92, 93]. By the end of 2022, over 20 PROTACs had reached the clinical stage globally [94]. The PROTACs ARV-110 (NCT03888612) and ARV-471 (NCT04072952), targeting the androgen receptor (AR) and estrogen receptor (ER), respectively, entered their first human trials in 2019. Both degraders have since progressed to phase II trials [95].

In a recent study by Gazorpak et al. (2023), a GR-targeting PROTAC named KH-103 was developed as a potential therapeutic tool for stress-related disorders. KH-103 facilitates the reversible and targeted degradation of GR, which has been demonstrated in several cell lines, including N2A, HEK293, and A549, as well as in hippocampal and cortical mouse primary neuronal cultures. Importantly, KH-103 selectively degraded GR without affecting MR levels, highlighting its specificity and potential clinical relevance [96].

Conceptually, PROTACs could directly degrade the malfunctioning GR, potentially restoring sensitivity to GC therapy. Unlike GR antagonists, which require continuous presence to block receptor activity with high concentrations, the GR degradation induced by a briefly applied low dose of a GR-PROTAC leads to sustained downregulation of the receptor. Another advantage is that, unlike GC synthesis inhibitors, the use of PROTACs can overcome compensatory mechanisms [97]. In the context of reversing central symptoms, several limitations of the technology require further exploration. In PROTAC design, the selection of the linker is crucial, as it impacts factors such as blood–brain barrier penetration, solubility, and overall pharmacokinetic properties [98]. Furthermore, the efficacy of PROTACs varies significantly across different cell types, contingent upon the specific substrate receptors involved in their associated E3 ubiquitin ligase complexes (e.g., cereblon (CRBN) [99], which vary between distinct cell types in the brain [100]. As PROTACs can be developed for the protein’s non-functional binding sites, genome-wide screens are being performed for optimal design [101, 102]. While still in the early stages of development, GR-PROTACs hold a great promise as a future therapeutic strategy.

## Strategies for normalizing decreased GR signaling

### GC supply

Optimizing the availability of GCs can be achieved by administering exogenous GCs. For example, Addison's disease, marked by adrenal insufficiency, is typically managed with GC replacement therapy; intriguingly, some individuals with concomitant depression have demonstrated improvement upon receiving single high dose GC [103]. For long-term therapy, adjusting doses and formulations is required to enhance GC absorption and distribution, while minimizing side effects [104, 105]. Time-dependent delivery methods, like chronotherapy or controlled-release formulations, may be particularly efficient by aligning administration with the body's circadian rhythms of GC production [106].

### GR agonists and modulators

Activation of the GR with synthetic agonists (e.g. Dexamethasone, Dex) is a gold standard for immunosuppression, antiallergic or anti-inflammatory therapy. Albeit Dex may display short-term beneficial effects in depression [107], its prolonged use replicates the excessive GR activation with detrimental consequences. The potential of novel GR modulators that spare the negative effects of GR stimulation [108, 109] remain to be demonstrated for beneficial outcomes on psychiatric symptoms.

### FKBP51 inhibitors

Antagonizing FKBP51 appears as a viable handle to the GR pathway, thanks to the powerful control exerted by FKBP51 on the GR transcriptional activity. Knocking out *FKBP5* has a protective effect on stress-coping behavior and stress endocrinology [110]. In silico analysis reveals that certain psychiatric drugs, including mirtazapine, sertraline, fluoxetine, and citalopram, exhibit pharmacological profiles conducive to serving as FKBP5 inhibitors [111]. The use of tacrolimus (FK506), an immunosuppressive agent that binds FKBP51 [112], is associated with various neuropsychiatric side effects, like psychosis [113, 114] and induction of depressive-like behavior in animal models [115]. These studies underscore the necessity of designing specific FKBP5 inhibitors for therapeutic use.

In a notable breakthrough, Gaali et al. (2015) developed selective antagonists of FKBP51 by induced fit SAFit1 and SAFit2, distinguished by their capacity to discriminate FKBP51 from FKBP52. Based on the crystal structure of the protein–ligand complex, the authors concluded that ligand binding induces conformational changes in FKBP51, enhancing the ligand affinity and specificity to FKBP51 [116]. Remarkably, the selective inhibition of FKBP51 by these innovative compounds was shown to elicit neurobiological effects with profound therapeutic implications [116, 117]. In N2A, SH-SY5Y neuroblastoma cell lines and primary hippocampal neurons, FKBP51 inhibition promoted neurite elongation, mimicking one of the main mechanisms proposed to mediate the beneficial action of antidepressants, i.e. restoring neuroplasticity [118]. Moreover, administration of SAFit2, which is more brain-permeable than SAFit1, reduced CORT levels at the circadian peak of glucocorticoid rhythm and reduced depressive-like behavior (forced swim test) in mice. These effects were associated with an enhancement in neuroendocrine feedback as SaFit2 suppressed the CORT levels both after Dex and corticotrophin-releasing factor (CRF) induction test [116]. Codagnone et al. (2021) demonstrated that mice chronically treated with SAFit2 exhibited reduced immobility in the forced swim test, decreased stress-induced behavior in the novelty-induced hypophagia test, and prevented stress-induced social avoidance after exposure to chronic stress models. Additionally, SAFit2 mitigated stress-induced behavior in the open field without impacting adult hippocampal neurogenesis [119]. Complementary in vitro assays revealed that SAFit2 promoted neurite outgrowth and neurogenesis, superior to brain-derived neurotrophic factor (BDNF), a key molecule postulated to mediate the action of antidepressants on neural plasticity [120]. A possible mechanistic explanation was provided by a study that found that stress or GR activation elicited FKBP5-dependent secretory autophagy and release of matrix metalloproteinase 9 (MMP-9) which cleaved pro-BDNF to its mature form (mBDNF), effects inhibited by SAFit [121]. Alternatively, SAFit may work by disrupting the interaction between FKBP51 and the GR, thereby restoring GR sensitivity and normalizing stress response systems [122].

Targeting FKBP51 may be a viable option also for other indications [123]. For example, Konig et al. (2018) observed that alcohol consumption decreased in mice when SAFit2 was applied [124]. Exploring the functional significance of the strong expression of FKBP51 in human muscle and adipose tissue, Balsevich et al., (2017) demonstrated that *Fkbp5* KO mice are resistant to high-fat diet-induced weight gain, have enhanced glucose tolerance, and have increased insulin signaling in skeletal muscle. The same results were obtained after administration of SAFit2 in terms of body weight regulation and glucose tolerance caused by *Fkbp5* deletion [125]. Additionally, SAFit2 improved the metabolic health of obese mice by improving glucose tolerance in high-fat diet conditions [125, 126]. The advancement of FKBP51-specific inhibitors progresses with mutagenic screens and protein engineering for improved ligand binding and reduced affinity to other immunophilins [127]. Future studies are needed to elucidate the therapeutic efficacy and safety profile of FKBP51 inhibitors in depression and stress-related disorders.

### FKBP51-PROTAC

Selective degradation of the FKBP51 protein can also be achieved with PROTAC technology. Recently, Geiger et al. (2024) reported SelDeg51, designed to target the scaffolding functions of FKBP51. Experiments carried out in HeLa cells showed that SelDeg51 enhanced the GR signaling, which was not observed with the inactive form of SelDeg51. Additionally, enhanced expression levels of *bona fide* GR target genes like *GILZ* and *FKBP5*, were induced in A259 cells treated simultaneously with GR agonist and SelDeg51. While the study demonstrated the potential of FKBP51-PROTAC technology in reversing GR suppression, the authors stated that the degradation rate of FKBP51 could be enhanced by further optimizations [128]. Once accomplished, evaluating SelDeg51 within the framework of stress-related disorders in vivo would be highly intriguing, as it represents a critical step toward advancing this compound to clinical trials.

## Future developments

### Combined therapies

The integration of various therapeutic approaches holds significant potential for optimizing treatment outcomes in mood disorders. When used in conjunction with antidepressants, FKBP51 inhibitors could amplify treatment efficacy by concurrently addressing hormonal and neurotransmitter imbalances and the negative effect of stress. For example, the addition of SAFit2 enhanced the efficacy of escitalopram, a broadly used SSRI, on stress-coping behavior, but diminished the drug impact in anxiety tests [129]. This data points to a need for detailed understanding of the role of GR-FKBP5 in discrete symptoms for tailoring personalized treatment options. Translational models combining deep phenotyping with molecular counterparts are now emerging with sufficient capacity to meet that challenge [130].

An important aspect of GR biology is its circadian dynamics. The intricate relationship between the circadian clock and the stress response systems is increasingly recognized as pivotal in understanding physiological rhythms and their impact on mental health [131]. Circadian clock genes regulate the rhythmic release of GCs, which in turn synchronize clocks in peripheral tissues [12, 132]. The concept of clock-based therapies offers a novel approach to addressing stress-related diseases [133, 134]. By leveraging GCs to stabilize circadian HPA axis regulation, individuals may gain protection against external stressors [135]. Intriguingly, transcriptional signature of fast antidepressant therapies, ketamine and sleep deprivation converge on the circadian gene network, a key process regulated by GR [136]. In the CNS, this effect may include the rescue of GR-mediated plasticity of neuronal connectivity [52, 137–139]. For the full advantage, technologies facilitating drug release at precise timings is crucial. Harnessing nanoparticle systems presents an avenue to regulate GC levels or modulate GR signaling with temporal precision [140, 141].

### Cell specific signaling

Understanding the GC effect with brain region- and cell-type-specific resolution is fundamental for advancing the field. Tools for conditional gene manipulation distinguished the role of GR and FKBP5 in distinct neural cell types [110, 142]. However, GCs exert a profound influence on glial cells. For example, systemic GR administration elicited a stronger transcriptional effect in striatal astrocytes than neurons [143]. Interestingly, the FKBP5 moderation of GR response exerted by variants associated with depression was also more prominent in astrocytes than neurons [144]. GR-induced pathways in astrocytes include crucial metabolic pathways, some specifically operated by these cells in the brain [145, 146]. The task of dissecting cell-specific transcriptional signatures of GR and its effects on the CNS biochemical profile is particularly challenging in the context of human genetics.

The solution is provided by methods based on human induced pluripotent stem cells (iPSCs) [147, 148]. The constantly expanding list of protocols enables to generation all major CNS cell types, with adequate brain regional profile for examining their biochemical profile and responsiveness to defined chemical stimuli. For example, the therapeutic outcomes in distinct cell types can be examined in cells derived from individuals profiled for *FKBP5* single nucleotide polymorphisms (SNPs), along with measures of relevant behavioral and physiological parameters, such as stress response and circadian rhythmicity.

## Limitations

GR modulation presents challenges, particularly due to the broad expression of GRs across various tissues, which can result in off-target effects, such as metabolic dysregulation and immune suppression [27]. Furthermore, sex differences play a significant role in GR signaling, leading to distinct effects in males and females [149]. Moreover, GC therapy can induce persistent epigenetic changes in paternal germ cells, affecting gene expression and behavioral traits in offspring across multiple generations. These findings underscore the challenge of adjusting GC therapies, as their transgenerational effects complicate the management of stress responses and long-term health outcomes [150–153].

Challenges in manipulating GR signaling include managing the delicate balance between therapeutic efficacy and the risk of withdrawal symptoms or exacerbating hypercortisolism. For instance, after adrenalectomy for conditions like Cushing’s syndrome, patients often require GC therapy to manage adrenal insufficiency due to prolonged HPA axis suppression. Tapering GC dose can lead to GC withdrawal symptoms such as fatigue, joint pain, muscle aches, depression, and anxiety, resulting from the body's prior dependence on elevated cortisol levels. Careful management of GC therapy is crucial to prevent adrenal insufficiency during recovery and ensure better patient outcomes [154], while inadequate regulation risks can also induce hypercortisolism [155].

Despite their promising advantages, PROTACs face several challenges. Their development is time-consuming, requiring extensive synthesis and medicinal chemistry. Off-target effects are a concern, as proteomics can reveal unexpected substrates and impact PROTAC selectivity. The hook effect at high concentrations can lead to unproductive dimers, complicating dosing [156]. Additionally, PROTACs have yet to prove effective across all protein classes or subcellular locations, especially for membrane-bound and organelle-located proteins [157]. Specific PROTACs targeting GR or FKBP5 may cause systemic side effects such as immune dysfunction or disrupted stress hormone regulation, highlighting the need for improved specificity and reduced toxicity in PROTAC design for stress-related disorders.

Precise mechanisms mediating the effects of GR modulators remain to be discovered. In this review, we focused on cell-specific actions in the CNS. However, GRs are also crucial in governing immune responses, metabolism, neuroprotection, or tissue repair in the periphery, each of which may contribute to ameliorating psychiatric symptoms [158, 159]. On the other hand, there is a growing body of evidence suggesting that restoring normal HPA axis function can have clinical benefits in treating depression [34, 160]. For example, while the GR antagonist mifepristone has demonstrated efficacy in alleviating depressive symptoms [34], the extent to which this outcome is attributable to modulation of the HPA axis versus direct GR-target tissue interactions remains uncertain.

## Conclusion

Treating stress-related disorders presents a multifaceted challenge, yet the strategic targeting of either hyperactivation or hypoactivation within the GR signaling pathway emerges as a pivotal avenue for therapeutic intervention. This approach requires a comprehensive understanding of the molecular mechanisms underlying stress-related disorders. For example, while overactivation of the HPA axis is commonly observed in MDD, some subpopulations exhibit HPA hypoactivity [161]. It becomes evident that a one-size-fits-all approach to treatment may not suffice. Instead, individual variations in genetic profile and patients’ history impacting stress responsiveness could hold the key to effectively treating mood disorders. Novel technologies facilitate tailored interventions to address specific molecular and physiological imbalances, ultimately improving the quality of life of individuals struggling with disease and offering hope for stable remission. In addition, developing reliable biomarkers for HPA axis function, GR sensitivity, and FKBP5 expression is crucial to guide the use of GR modulators. This need underscores the importance of advancing such biomarkers to ensure personalized and effective treatment for stress-related disorders.

## Data Availability

Data sharing is not applicable to this article as no datasets were generated or analyzed during the current study.

## Abbreviations

AR: Androgen receptor
BDNF: Brain-derived neurotrophic factor
CNS: Central nervous system
CORT: Corticosterone
CRBN: Cereblon
CRF: Corticotropin-releasing factor
Dex: Dexamethasone
ER: Estrogen receptor
FKBP51: FK506-binding protein 51
FKBP52: FK506-binding protein 52
GC: Glucocorticoid
GR: Glucocorticoid receptor
GRE: GC response element
HPA: Hypothalamic–pituitary–adrenal
iPSCs: Induced pluripotent stem cells
IR: Inverted repeats
KO: Knockout
LYTAC: Lysosome- targeting chimera
mBDNF: Mature brain-derived neurotrophic factor
MDD: Major depressive disorder
MMP-9: Matrix metalloproteinase 9
MR: Mineralocorticoid receptor
nGRE: Negative GC response element
PROTAC: Proteolysis targeting chimera
PTSD: Post-traumatic stress disorder
SNIPERs: Specific and non-genetic inhibitors of apoptosis protein-dependent protein erasers
SNPs: Single nucleotide polymorphisms
SSRI: Serotonin reuptake inhibitor
TF: Transcription factor
